# HbA1c performs well in monitoring glucose control even in populations with high prevalence of medical conditions that may alter its reliability: the OPTIMAL observational multicenter study

**DOI:** 10.1136/bmjdrc-2021-002350

**Published:** 2021-09-17

**Authors:** Anxious J Niwaha, Lauren R Rodgers, Rosamund Greiner, Priscilla A Balungi, Raymond Mwebaze, Timothy J McDonald, Andrew T Hattersley, Beverley M Shields, Moffat J Nyirenda, Angus G Jones

**Affiliations:** 1 Institute of Biomedical and Clinical Science, College of Medicine and Health, University of Exeter, Exeter, UK; 2 NCD Theme, MRC/UVRI and LSHTM Uganda Research Unit, Entebbe, Uganda; 3 Institute of Health Research, University of Exeter Medical School, Exeter, UK; 4 Department of Medicine, St. Francis Hospital Nsambya, Kampala, Uganda; 5 NCD Epidemiology, London School of Hygiene & Tropical Medicine, London, UK

**Keywords:** CGM, HbA1c, monitoring, diabetes mellitus, type 2

## Abstract

**Introduction:**

The utility of HbA1c (glycosylated hemoglobin) to estimate glycemic control in populations of African and other low-resource countries has been questioned because of high prevalence of other medical conditions that may affect its reliability. Using continuous glucose monitoring (CGM), we aimed to determine the comparative performance of HbA1c, fasting plasma glucose (FPG) (within 5 hours of a meal) and random non-fasting glucose (RPG) in assessing glycemic burden.

**Research design and methods:**

We assessed the performance of HbA1c, FPG and RPG in comparison to CGM mean glucose in 192 Ugandan participants with type 2 diabetes. Analysis was undertaken in all participants, and in subgroups with and without medical conditions reported to affect HbA1c reliability. We then assessed the performance of FPG and RPG, and optimal thresholds, in comparison to HbA1c in participants without medical conditions thought to alter HbA1c reliability.

**Results:**

32.8% (63/192) of participants had medical conditions that may affect HbA1c reliability: anemia 9.4% (18/192), sickle cell trait and/or hemoglobin C (HbC) 22.4% (43/192), or renal impairment 6.3% (12/192). Despite high prevalence of medical conditions thought to affect HbA1c reliability, HbA1c had the strongest correlation with CGM measured glucose in day-to-day living (0.88, 95% CI 0.84 to 0.91), followed by FPG (0.82, 95% CI 0.76 to 0.86) and RPG (0.76, 95% CI 0.69 to 0.81). Among participants without conditions thought to affect HbA1c reliability, FPG and RPG had a similar diagnostic performance in identifying poor glycemic control defined by a range of HbA1c thresholds. FPG of ≥7.1 mmol/L and RPG of ≥10.5 mmol/L correctly identified 78.2% and 78.8%, respectively, of patients with an HbA1c of ≥7.0%.

**Conclusions:**

HbA1c is the optimal test for monitoring glucose control even in low-income and middle-income countries where medical conditions that may alter its reliability are prevalent; FPG and RPG are valuable alternatives where HbA1c is not available.

**Significance of this study**

**What is already known about this subject?**

- HbA1c is the gold standard for monitoring glycemic control.
- The value of HbA1c measurement among populations living in low-resource settings has been questioned because of high prevalence of other medical conditions that may affect test reliability, such as hemoglobinopathies or anemia.

**What are the new findings?**

- HbA1c is the overall best measure of glycemic burden, despite high prevalence of other medical conditions that may affect its accuracy (eg, anemia, hemoglobinopathies).
- Fasting plasma glucose (FPG) and random non-fasting glucose (RPG) were strongly correlated with continuous glucose monitoring (CGM) glucose and HbA1c, and had reasonable sensitivity and specificity to detect poor glycemic control.
- The difference in performance between these tests is modest.

**How might these results change the focus of research or clinical practice?**

- HbA1c is the optimal laboratory method for assessing glycemic control, even in populations with high prevalence of conditions reported to affect test reliability.
- FPG and RPG measurements correlate strongly with both CGM and HbA1c, perform reasonably well in identifying poor glycemic control and can therefore be used when HbA1c is unavailable.

## Introduction

Diabetes is a global problem disproportionately affecting low-income and middle-income countries (LMICs), with 80% of the global 463 million people with diabetes living in LMICs.^1^ Unlike high-income countries, diabetes healthcare in LMICs is underfunded^1^ and lacks quality, pragmatic and contextualized guidelines.^2^ As such, LMICs are heavily impacted by high rates of poorly controlled glucose levels,^3–5^ and subsequently, high rates of diabetes-related complications and poor quality of life among people living with diabetes.

Monitoring glycemic control is essential to allow appropriate titration of medication and improve outcomes among patients with diabetes, but regular monitoring can be challenging in LMICs. In high-income countries, HbA1c (glycosylated hemoglobin) is the recommended measure used for assessing glucose control and titrating medications, often supported by home glucose capillary or interstitial glucose monitoring.^6 7^ However, financial constraints mean that the monitoring of diabetes and decisions to intensify treatment in much of the low-income regions are predominantly based on testing of a single glucose measure.^8^ This is because HbA1c testing is not routinely available in most centers,^8^ and HbA1c is often too expensive for the majority of patients.^9^ Even where testing is available, there has been substantial concern that HbA1c measurement may be unreliable in LMIC populations,^10–12^ due to high prevalence of hemoglobinopathies such as sickle cell and thalassemia, and other medical conditions that might affect test reliability including anemia and malaria.^13^ Home glucose monitoring is not well funded by healthcare systems in LMICs and is beyond the financial means and literacy skills of a large proportion of those who have diabetes.^8 14^

International organisations recommend the use of plasma glucose for monitoring glycemic control in developing countries where HbA1c services are not readily available.^15^ However, assessment of glycemic control in such settings is normally after long walks by the patients to attend a centralized clinic every 2–3 months, coupled with prolonged fasting and long waiting times.^16^ As such, many clinicians rely on a random glucose without the requirement to fast to assess glycemia.^16^ While these tests have been compared with HbA1c in the LMIC setting,^17 18^ given the limitations of HbA1c itself in these populations, its performance as a measure of average glucose is unclear. Continuous glucose monitoring (CGM) offers the opportunity of measuring glucose in day-to-day living over a period of days to weeks and is widely used in high-income countries and some LMICs.

In the OPTIMAL study, we aimed to compare, in an African population with type 2 diabetes, the accuracy of fasting plasma glucose (FPG), random non-fasting plasma glucose (RPG), and HbA1c in comparison to CGM as an independent measure of glycemic control, and assess the impact of other medical conditions that may affect HbA1c reliability to monitor glycemia in people with established diabetes.

## Methods

### Study population

Participants were recruited from diabetes clinics in Masaka Regional Referral Hospital (rural, public) and St. Francis Hospital Nsambya (urban, private not-for-profit) in Uganda and met the following inclusion criteria: a clinical diagnosis of type 2 diabetes, diagnosed at the age of 18 years and above, more than 12 months’ diabetes duration, no initial insulin requirement for at least 1 year since the time of diagnosis, no change in glucose-lowering therapy 3 months prior, and able to give informed consent. Participants who were pregnant or judged by their clinician to need an immediate change in glucose-lowering medication were excluded from recruitment.

### Study visits

Participants were scheduled for three visits. The overview of the study design is presented in online supplemental figure S1.

10.1136/bmjdrc-2021-002350.supp1 Supplementary data

At the baseline visit, participants came to the clinic in a non-fasted state. Following assessment of clinical features and demographics, non-fasting (within 5 hours of a meal) random blood sample was collected for measurement of RPG, HbA1c, full blood count, lipid profile, renal function and assessment of hemoglobin variants. CGM was carried out using the Freestyle Libre Pro Flash Glucose Monitoring System (Abbott Laboratories, Illinois, USA), a professional CGM device which records interstitial glucose every 15 min for up to 2 weeks. Freestyle Libre Pro is blinded, meaning data could not be viewed by the wearer.

All participants returned in a fasted state (at least 8 hours) in the second week of CGM between days 7 and 10 from the baseline visit, and for their final visit, between days 12 and 14 from the baseline visit, in a non-fasted state (within 5 hours of a meal). At both of these visits, CGM data were downloaded and a venous blood sample was collected for measurement of HbA1c and RPG (visits 1 and 3) and FPG (visit 2). The study was carried out in accordance with the 2008 revised principles of the Declaration of Helsinki and all participants provided informed consent before study activities.

### Patient and public involvement (PPI)

Patients were involved in prioritization of the research question. Patients were not involved in the design and conduct of the study. However, they were central to dissemination of the results by choosing to have some of the results sent to their respective clinicians, and will continue to be involved in ongoing study dissemination.

### Laboratory procedures

Blood samples for glucose measurement were collected in a vacutainer with sodium fluoride (NaF), centrifuged and separated into two cryovials (aliquots) immediately and kept in an icebox at 4°C–8°C before being transported to the central laboratory for immediate testing (within 8 hours of collection). Whole blood samples for full blood count and HbA1c were collected in vacutainers containing EDTA. All analytical measurements were performed at the Central Biochemistry and Clinical Diagnostic Laboratory Services (CDLS) laboratory at the MRC/UVRI & LSHTM Research Unit Entebbe Uganda. Laboratory analyses were performed on a Roche Cobas 6000 analyzer (Hitachi High Technologies, Tokyo, Japan). Plasma glucose was measured by the glucokinase method. HbA1c was also measured on Cobas 6000 by the immunoassay technique, calibrated to the International Federation of Clinical Chemistry. Hemoglobinopathies (sickle cell trait and hemoglobin C (HbC)) were assessed by Hb electrophoresis.

### CGM measures

Raw glucose readings were downloaded from the Libreview software and CGM summary variables (including mean CGM glucose) were calculated using R V.3.6.1. Sensor data were considered for analysis if the total duration of CGM wear was at least 5 days.

For CGM validation, we matched plasma FPG at visit 2 with a nearest CGM glucose value within 15 min. We then determined the relationship between the plasma glucose and the CGM glucose value using Bland-Altman analysis to assess the degree of bias and levels of agreement between the sensor and plasma glucose.

### Statistical analysis

Data were analyzed using Stata V.16.1 (StataCorp LLC, USA).

#### Comparison of glucose and HbA1c measures with CGM measured glucose in daily living

We assessed the strength of the relationship between CGM assessed mean glucose over 2 weeks and each of FPG, RPG and HbA1c using Pearson’s correlation coefficients and linear regression. Analysis was based on RPG and HbA1c tests performed on the last visit (visit 3), unless not available, in which case values from visit 1 were used instead (n=9). To assess the impact of other medical conditions (anemia, hemoglobinopathies, and renal impairment) on HbA1c reliability, we subdivided the cohort into those without medical conditions that may alter HbA1c reliability and those with medical conditions that may alter HbA1c reliability. HbA1c performance in comparison to CGM was assessed in all participants regardless of comorbidities, and by presence or absence of medical conditions thought to affect test performance (see below). Equivalent thresholds for predicting suboptimal glycemic control (defined as CGM glucose values ≥8 and ≥10 mmol/L) were derived from linear regression equations. We compared the performance of RPG and FPG and HbA1c to identify participants with CGM glucose values ≥8 and ≥10 mmol/L using receiver operating characteristic curve analysis, and assessed the sensitivity, specificity and positive/negative predictive values of these tests using the equivalent cut-offs derived from linear regression equations.

#### Comparison of FPG and RPG measurement with HbA1c

As HbA1c is the measure which has been robustly validated against clinical outcomes, we performed additional analysis, where we assessed the strength of the relationship between HbA1c and each of the FPG and RPG tests in the absence of medical conditions that might affect HbA1c reliability. Participants were considered to have no other medical conditions that may affect HbA1c reliability if they met the following characteristics: no hemoglobinopathies (sickle cell trait and HbC), absence of anemia (Hb in women ≥120 g/L, men ≥130 g/L),^19^ and no renal impairment (estimated glomerular filtration rate (eGFR) ≥60 mL/min/1.73 m^2^). In participants without these medical conditions, we determined diagnostic performance of the glucose tests for suboptimal glucose control defined by HbA1c at the following thresholds: HbA1c ≥48 mmol/mol (6.5%), ≥53 mmol/mol (7.0 %), 58 mmol/mol (7.5%), 64 mmol/mol (8.0%), 69 mmol/mol (8.5%) and 75 mmol/mol (9.0%). Equivalent thresholds of FPG and random glucose for predicting suboptimal glycemic control were obtained by linear regression analysis.

## Results

### Baseline characteristics

A total of 213 adults were enrolled in the study. Of these participants, 9.86% (21/213) were excluded for insufficient data. Characteristics of excluded participants were broadly similar to those included in analysis, as shown in online supplemental table 1. Out of 213 participants, 192 had sufficient data for inclusion in the final analysis (see flow chart: online supplemental figure S2). The median CGM duration was 14 (IQR 13–14) days. Participant characteristics are presented in table 1. Average glycemic control was poor with a median (IQR) HbA1c of 67 (52.0–90.0) mmol/mol (8.3% (6.9–10)). The other medical conditions that may affect HbA1c reliability were common, occurring in 32.8% (63/192) of participants, of whom 9.4% (18/192) had anemia, 22.4% (43/192) had hemoglobinopathies (sickle cell trait (n=43) and/or hemoglobin AC (HbAC) (n=1)), and 6.3% (11/190) had renal impairment (eGFR <60 mL/min/1.73 m^2^). Characteristics according to absence or presence of medical conditions that may affect HbA1c reliability are shown in online supplemental table 2.

10.1136/bmjdrc-2021-002350.supp2 Supplementary data

**Table 1:** Participant characteristics (N=192)

	Median (IQR) for continuous variables, % (n) for proportions
Clinical	
Female, n (%)	58.3 (112/192)
Age, years	56 (50–63)
Duration of diabetes, years	6 (3–10)
BMI, kg/m^2^	26.8 (24.0–30.5)
Current management, n (%)	
Metformin only	15.6 (30/192)
SU (±metformin)*	57.3 (110/192)
Insulin (±other diabetes drug)†	26.0 (50/192)
Diet‡	1.0 (2/192)
Glycemia	
CGM glucose, mmol/L	8.6 (6.8–12.3)
HbA1c, %	8.3 (6.9–10.0)
HbA1c, mmol/mol	67 (52.0–90.0)
FPG, mmol/L	8.2 (6.1–11.4)
RPG, mmol/L	13.5 (8.8–17.2)
Other laboratory	
Hb (g/L)	14.2 (13.2–15.0)
Anemia§	9.4% (18/192)
Hemoglobinopathies, n (%)¶	22.4% (43/192)
eGFR	111.5 (92.3–121.0)
Renal impairment, n (%)	6.3% (12/192)

*Sulfonylureas with or without metformin.

†Insulin with or without any oral therapy.

‡Two participants were on non-pharmacological management (diet) only.

§ Anemia was defined as a Hb of <120 g/L in women and <130 g/L in men.

¶Hemoglobinopathies was defined as the presence of sickle cell trait (HbAS) or HbAC.

BMI, body mass index; CGM, continuous glucose monitoring; eGFR, estimated glomerular filtration rate; FPG, fasting plasma glucose; Hb, hemoglobin; HbAC, hemoglobin AC; HbA1c, glycosylated hemoglobin; RPG, random non-fasting plasma glucose.

### FPG and CGM glucose are highly correlated

FPG and CGM glucose (closest value, within 15 min) were highly correlated (Pearson’s r=0.97, 95% CI 0.96 to 0.98). CGM values showed a modest bias toward lower glucose than FPG, with CGM values mean 1.3 (95% CI 1.1 to 1.5) mmol/L lower—this was consistent across the range of glycemic control (online supplemental figure S3).

### HbA1c has the strongest relationship with CGM glucose in an African population, even in participants with comorbidities thought to alter HbA1c reliability

**Figure 1:**
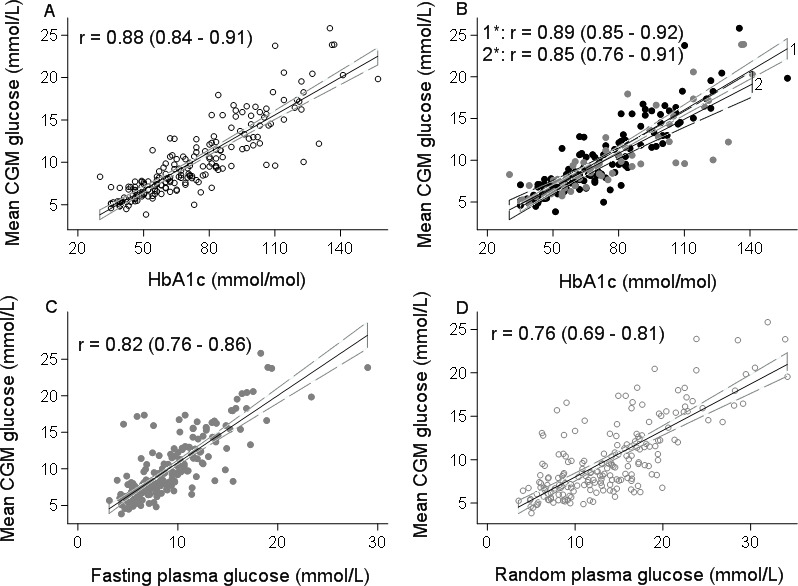
Comparison of (A) HbA1c (glycosylated hemoglobin) of the overall sample population and (B) HbA1c without (1; black circles) and with (2; gray circles) conditions thought to alter HbA1c reliability with mean continuous glucose monitoring (CGM) glucose. Comparison of (C) fasting plasma glucose (FPG) and (D) random non-fasting plasma glucose (RPG) with mean CGM glucose. Solid straight line denotes the line of best fit and the dashed lines represent the 95% CI. The Pearson’s correlation coefficient (r) and 95% CIs are shown for each graph. Conditions thought to alter HbA1c reliability include hemoglobinopathies including sickle cell trait and hemoglobin AC (HbAC), anemia, and renal impairment.

The relationship between HbA1c, FPG and RPG tests and average CGM glucose is shown in figure 1. There was a strong correlation between all the three tests and mean CGM glucose. HbA1c had the strongest correlation (0.88; 95% CI 0.84 to 0.91), followed by FPG (0.82; 95% CI 0.76 to 0.86) and RPG (0.76; 95% CI 0.69 to 0.81). The derived linear equations for estimating mean glucose from HbA1c, FPG and RPG among patients with diabetes are shown in online supplemental table 3. The diagnostic performances of HbA1c, FPG and RPG tests for diagnosing suboptimal glucose control (defined by illustrative mean CGM thresholds of 8 and 10 mmol/L) are shown in table 2. There was a very modest loss of diagnostic performance using FPG compared with HbA1c, at equivalent thresholds. HbA1c was the most sensitive and specific test followed by FPG.

**Table 2:** Ability of HbA1c, FPG and RPG to define suboptimal glucose control using CGM thresholds <8 and <10 mmol/L

CGM cut-off	Test	N	AUROC (95% CI)	Optimal threshold	Sensitivity (95% CI)	Specificity (95% CI)	Correctly classified	PPV (95% CI)	NPV (95% CI)
≥8.0	HbA1c	191	0.95 (0.92 to 0.98)	≥62 mmol/mol	90.2 (83.1 to 95.0)	83.5 (73.5 to 90.9)	87.4	88.6 (81.3 to 93.8)	85.7 (75.9 to 92.6)
	FPG	191	0.90 (0.86 to 0.95)	≥7.6 mmol/l	84.8 (76.8 to 90.9)	81.0 (70.6 to 89.0)	83.3	86.4 (78.5 to 92.2)	79.0 (68.5 to 87.3)
	RPG	192	0.82 (0.77 to 0.88)	≥11.6 mmol/l	78.6 (69.8 to 85.8)	64.6 (53.0 to 75.0)	72.8	75.9 (67.0 to 83.3)	68.0 (56.2 to 78.3)
≥10.0	HbA1c	191	0.94 (0.90 to 0.97)	≥72 mmol/mol	88.9 (79.3 to 95.1)	84.9 (77.2 to 90.8)	86.4	78.0 (67.5 to 86.4)	92.7 (86.0 to 96.8)
	FPG	191	0.90 (0.85 to 0.95)	≥9.1 mmol/l	83.6 (73.0 to 91.2)	83.1 (75.0 to 89.3)	83.3	75.3 (64.5 to 84.2)	89.1 (81.7 to 94.2)
	RPG	192	0.85 (0.79 to 0.91)	≥13.8 mmol/l	84.7 (74.3 to 92.1)	72.3 (63.3 to 80.1)	77.0	64.9 (54.4 to 74.5)	88.7 (80.6 to 94.2)

The units used are as follows: HbA1c—mmol/mol and mmol/L for fasting and random non-fasting glucose.

AUROC, area under receiver operating characteristic curve; CGM, continuous glucose monitoring; FPG, fasting plasma glucose; HbA1c, glycosylated hemoglobin; NPV, negative predictive value; PPV, positive predictive value; RPG, random non-fasting plasma glucose.

HbA1c maintained the strongest relationship with CGM glucose even in those with other medical conditions that might affect HbA1c reliability (figure 1). In those with and without conditions that might affect HbA1c reliability, the relationship between CGM glucose and HbA1c was similar, with no difference in correlation (0.85; 95% CI 0.76 to 0.91) versus (0.89; 95% CI 0.85 to 0.92) (figure 1) and the difference in linear regression slopes was modest (mean CGM glucose=0.14*HbA1c–0.02 and 0.16*HbA1c–1.07 with and without conditions that may affect HbA1c reliability, respectively) (online supplemental table 3). This was also similar when examining only those with hemoglobinopathy (r=0.90, 95% CI 0.82 to 0.94, n=42, supplementary figure S4).

### FPG and RPG have broadly similar diagnostic performance in identifying patients with poor glycemia control

**Figure 2:**
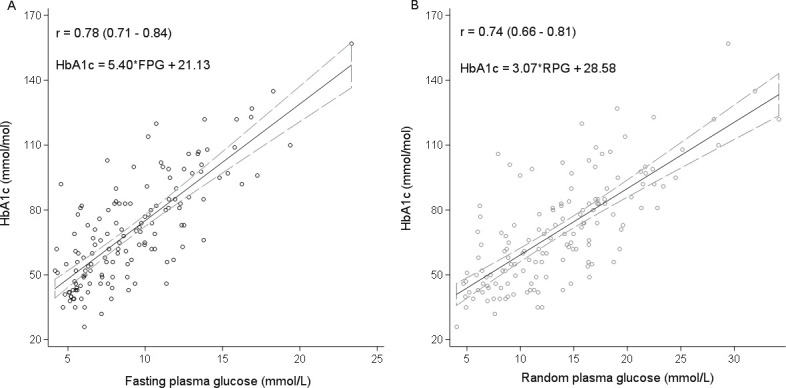
(A, B) Comparison of fasting plasma glucose (FPG) and random non-fasting plasma glucose (RPG) with HbA1c (glycosylated hemoglobin) in participants without conditions thought to alter HbA1c reliability. Solid straight line denotes the line of best fit and the dashed lines represent the 95% CI. The Pearson’s correlation coefficient (r) and 95% CIs are shown for each graph. Conditions thought to alter HbA1c reliability include hemoglobinopathies including sickle cell trait and hemoglobin AC (HbAC), anemia, and renal impairment.

Among participants without conditions thought to alter HbA1c reliability (including hemoglobinopathies, anemia and renal impairment), RPG and FPG had similar correlation with HbA1c (0.74; 95% CI 0.65 to 0.80) and (0.78; 95% CI 0.71 to 0.84), respectively (figure 2). The equivalent thresholds and diagnostic performances of FPG and RPG for predicting HbA1c defined suboptimal glucose control (at different HbA1c thresholds), restricted to those without conditions thought to alter HbA1c reliability, are shown in table 3. FPG and RPG had very similar performance in identifying those with suboptimal glycemic control (table 3). For the widely used HbA1c target of 7.0%, the AUC ROC for these tests was similar (FPG 0.76, RPG 0.77). At their respective optimal thresholds (FPG ≥7.1 mmol/L and RPG ≥10.5 mmol/L), the tests had a similar sensitivity (FPG −81.0, 95% CI 71.9 to 88.2 vs RPG −81.6, 95% CI 72.7 to 88.5) and specificity (FPG −71.4, 95% CI 55.4 to 84.3 vs RPG −72.1, 95% CI 56.3 to 84.7) for identifying suboptimal glycemic control. The linear equations for estimating HbA1c from FPG and RPG among patients with diabetes were HbA1c (mmol/mol)=5.40*FPG+21.3 and HbA1c=3.07*RPG+28.58, respectively, for patients without comorbidities thought to alter HbA1c (online supplemental table 4).

**Table 3:** Ability of FPG and RPG to predict suboptimal glucose control among patients with type 2 diabetes without medical conditions thought to alter HbA1c reliability using different HbA1c thresholds

HbA1c cut-off	Test	N	AUROC (95% CI)	Equivalent threshold (mmol/L)	Sensitivity (95% CI)	Specificity (95% CI)	PPV (95% CI)	NPV (95% CI)	Correctly classified (%)
48 (6.5%)	FPG	142	0.84 (0.77 to 0.92)	6.6	79.5 (70.8 to 86.5)	73.3 (54.1 to 87.7)	91.8 (84.4 to 96.4)	48.9 (33.7 to 64.2)	78.2
	RPG	145	0.86 (0.80 to 0.92)	9.6	79.1 (70.6 to 86.1)	71.0 (52.0 to 85.8)	91.0 (83.6 to 95.8)	47.8 (32.9 to 63.1)	77.4
53 (7.0%)	FPG	142	0.87 (0.81 to 0.93)	7.1	81.0 (71.9 to 88.2)	71.4 (55.4 to 84.3)	87.1 (78.5 to 93.2)	61.2 (46.2 to 74.8)	78.2
	RPG	145	0.88 (0.83 to 0.94)	10.5	81.6 (72.7 to 88.5)	72.1 (56.3 to 84.7)	87.5 (79.2 to 93.4)	62.0 (47.2 to 75.3)	78.8
58 (7.5%)	FPG	142	0.85 (0.79 to 0.91)	7.7	76.7 (66.6 to 84.9)	76.9 (63.2 to 87.5)	85.2 (75.6 to 92.1)	65.6 (52.3 to 77.3)	76.8
	RPG	145	0.84 (0.77 to 0.90)	11.4	78.5 (68.8 to 86.3)	71.7 (57.7 to 83.2)	83.0 (73.4 to 90.1)	65.5 (51.9 to 77.5)	76.0
64 (8.0%)	FPG	142	0.86 (0.80 to 0.92)	8.4	74.0 (62.8 to 83.4)	81.5 (70.0 to 90.1)	82.6 (71.6 to 90.7)	72.6 (60.9 to 82.4)	77.5
	RPG	145	0.84 (0.78 to 0.90)	12.4	78.8 (68.2 to 87.1)	80.3 (68.7 to 89.1)	82.9 (72.5 to 90.6)	75.7 (64.0 to 85.2)	79.5
69 (8.5%)	FPG	142	0.85 (0.79 to 0.91)	9.0	73.5 (61.4 to 83.5)	83.8 (73.4 to 91.3)	80.6 (68.6 to 89.6)	77.5 (66.8 to 86.1)	78.9
	RPG	145	0.85 (0.79 to 0.91)	13.3	76.1 (64.5 to 85.4)	78.7 (67.7 to 87.3)	77.1 (65.6 to 86.3)	77.6 (66.6 to 86.4)	77.4
75 (9.0%)	FPG	142	0.85 (0.78 to 0.92)	9.6	74.1 (60.3 to 85.0)	80.7 (70.9 to 88.3)	70.2 (56.6 to 81.6)	83.5 (73.9 to 90.7)	78.2
	RPG	145	0.85 (0.78 to 0.92)	14.4	75.0 (61.6 to 85.6)	77.8 (67.8 to 85.9)	67.7 (54.7 to 79.1)	83.3 (73.6 to 90.6)	76.7

AUROC, sensitivity, specificity, % correctly classified, PPV and NPV are given for the respective optimal thresholds of the test. This was restricted to HbA1c where there were no conditions thought to alter HbA1c reliability like anemia, sickle cell traits, and renal impairment.

The units used are mmol/L for fasting and random non-fasting glucose.

AUROC, area under receiver operating characteristic curve; FPG, fasting plasma glucose; HbA1c, glycosylated hemoglobin; NPV, negative predictive value; PPV, positive predictive value; RPG, random non-fasting plasma glucose.

## Discussion

The international guidelines recommend HbA1c for monitoring glycemic control and blood glucose test where HbA1c is unavailable. Despite this guidance, there remains concerns about the accuracy of HbA1c in populations with high frequency of other medical conditions that may alter its reliability. In this study, we used CGM to compare the accuracy of HbA1c, FPG and RPG tests in assessing glycemic control among patients with diabetes under conditions of everyday life in low-resource settings. The prevalence of other medical conditions that may alter HbA1c reliability was remarkably high. However, we found that HbA1c remained the most accurate test of average glucose control, despite the high prevalence of hemoglobinopathies, anemia and renal impairment. Similarly, FPG and RPG demonstrated reasonable accuracy as measures of average glycemic control, providing confidence that glucose tests provide a good measure of glycemia where HbA1c is not available. Furthermore, the very modest loss of diagnostic test performance using RPG provides some reassurance for use of this test in situations where a RPG is the only or most practical measure available.

In the current study, we have compared FPG, RPG and HbA1c in the same study and more importantly against an independent measure of day-to-day glycemic burden. CGM was used as an independent marker of glycemic burden to allow assessment of the relative performance of HbA1c, FPG and RPG in assessing glycemic burden. This is a major strength of our analysis in contrast to previous studies which have compared between measures such as HbA1c and FPG, with no independent comparison. Further, we assessed performance of HbA1c in the presence of other medical conditions that may alter its effect. This gave us the opportunity to assess the overall impact on HbA1c reliability.

However, the present study has some limitations that should be taken into consideration. First, although CGM was the best available option for direct measurement of glucose in day-to-day living and allowed us to compare the relative performance of HbA1c and glucose tests, it should be noted that glycemia was measured using a CGM sensor over median 14 (IQR 13–14) days and yet HbA1c estimates glycemia over a longer duration.^20^ Second, we used HbA1c immunoassay, one of the most widely used HbA1c assays, particularly in low-resource settings. However, our results for the performance might not apply to other HbA1c assay types, which are known to have different susceptibility to the effects of hemoglobinopathies.^21^ Furthermore, although we screened for a number of potential comorbidities thought to alter HbA1c, with the available sample size and very modest subgroup numbers, we were unable to do further subgroup analyses to assess the impact of other individual underlying non-glycemic conditions.^22^ In addition, the impact of glucose-6-phosphate dehydrogenase variants, another common condition that may affect HbA1c results reliability, was not assessed.^23^

Our results showing a strong relationship between HbA1c and mean glucose from CGM are consistent with studies that have compared these two measures in high-income settings. The Diabetes Control and Complications Trial (DCCT) of participants in the USA with type 1 diabetes showed a strong relationship between the mean plasma glucose and HbA1c with a Pearson correlation (r) of 0.82.^24^ Similarly, results from the ADAG (A1c Derived Average Glucose) study, which included 507 participants with and without diabetes predominantly from the USA and Europe, and excluded participants with other medical conditions thought to alter HbA1c reliability, showed HbA1c and mean glucose were closely correlated (r=0.89, p<0.0001).^25^ Our similar results (r=0.88) in an African population, and without exclusion of participants with analytical concerns for HbA1c measurement, is reassuring for the use of HbA1c testing in this region.

Our results are broadly consistent with previous studies that have reported the relationship between glucose tests and HbA1c. El-Kebbi *et al* showed, in 1827 predominantly African–American living in the USA, that RPG collected 1–4 hour post meal was correlated strongly with HbA1c, although in this predominantly insulin-treated population, the correlation (r=0.63) was lower than observed in our study (0.74).^26^ In a study that compared both FPG and RPG to HbA1c among 1000 patients with diabetes living in India, FPG showed a better correlation with HbA1c than RPG (0.739 vs 0.601).^27^ In contrast, in studies where a fixed post meal time point was used, RPG was a slightly better correlate of HbA1c than FPG.^18^ Unfortunately, studies comparing performances of glucose tests against HbA1c in Africa are very few, with small sample sizes, and in these studies, the impact of common medical conditions that may alter HbA1c reliability was not assessed.^17 28^

Our data suggest that there is a high prevalence of other medical conditions that may alter HbA1c reliability justifying the questioning of HbA1c utility. However, even with these comorbidities, HbA1c, when measured with an immunoassay method, correlated strongly with mean glucose, outperforming glucose measures, and only displayed a modest improvement when patients with comorbidities were excluded. This suggests that HbA1c remains the optimal laboratory method of monitoring glucose burden even where prevalence of conditions that may affect its reliability is high. The strong correlation of HbA1c with glucose despite the prevalence of other medical conditions that may alter HbA1c reliability deserves further exploration. However, there are some reasons why the impact of these conditions on HbA1c reliability may be modest in this setting. First, in line with the National Glycohemoglobin Standardization Program (NGSP) recommendation, modern HbA1c immunoassays are not directly affected by the presence of hemoglobin variants like HbAS.^21^ Second, while comorbidities that affect red cell life will alter the accuracy of any HbA1c method, the predominant hemoglobinopathy in our study population was HbAS (sickle cell) trait, and previous research has been conflicting as to whether this meaningfully alters red cell lifespan.^29^

While our results support the use of HbA1c (where available) rather than glucose measures in LMIC populations, the small subgroup numbers in our study limited the power to definitively determine the impact of some of these comorbidities on HbA1c performance. To accurately determine the impact of individual comorbidities, larger multinational studies involving other regions in Africa and LMICs with enrichment for these comorbidities would be needed. Furthermore, while our data show that HbA1c (measured using an immunoassay method) has the closest relationship with average glucose, even with comorbidities, it is possible that the overall relationship between glucose and HbA1c is different in this population, therefore the thresholds used internationally are not appropriate, and bespoke HbA1c thresholds are needed for different populations. This further underscores the need for much larger studies, ideally incorporating risk of microvascular complications, to determine whether the HbA1c targets used internationally are appropriate for LMIC populations.

In conclusion, our results suggest that HbA1c is the optimal test for monitoring glucose control even in LMICs where medical conditions that may alter its reliability are prevalent; FPG and RPG are valuable alternatives where HbA1c is not available.

## Data Availability

Data are available upon reasonable request. Data analysed in this study are not available for public use but to researchers upon reasonable request from the corresponding author.
