# Supplementary material for: HbA1c performs well in monitoring glucose control even in populations with high prevalence of medical conditions that may alter its reliability: the OPTIMAL observational multicenter study
Source: BMJ Open Diabetes Res Care. 2021 Sep 17;9(1):e002350. doi: 10.1136/bmjdrc-2021-002350 (PMC8451306; doi:10.1136/bmjdrc-2021-002350)
Supplement: Supplementary data [file bmjdrc-2021-002350supp001.pdf]

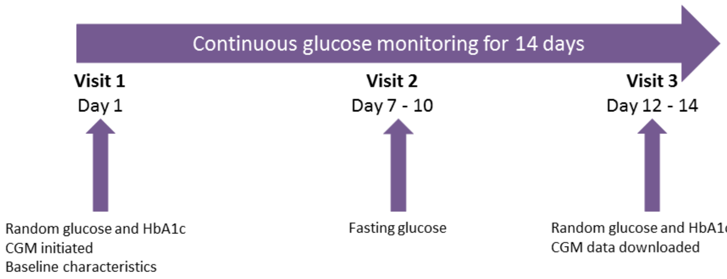

Figure S1: Overview of the study design

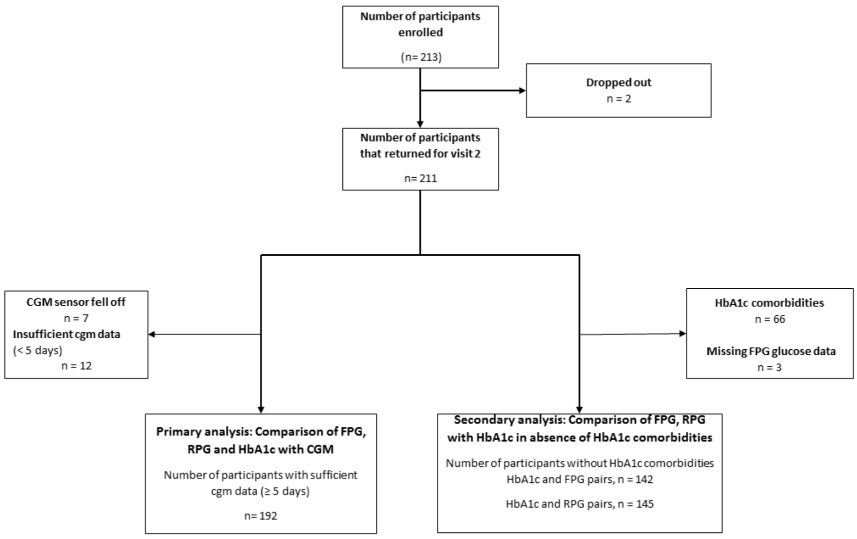

Figure S2: Participant flow chart. Conditions thought to alter HbA1c reliability (HbA1c comorbidities) include haemoglobinopathies (sickle cell trait and HbAC), anaemia, and renal impairment.

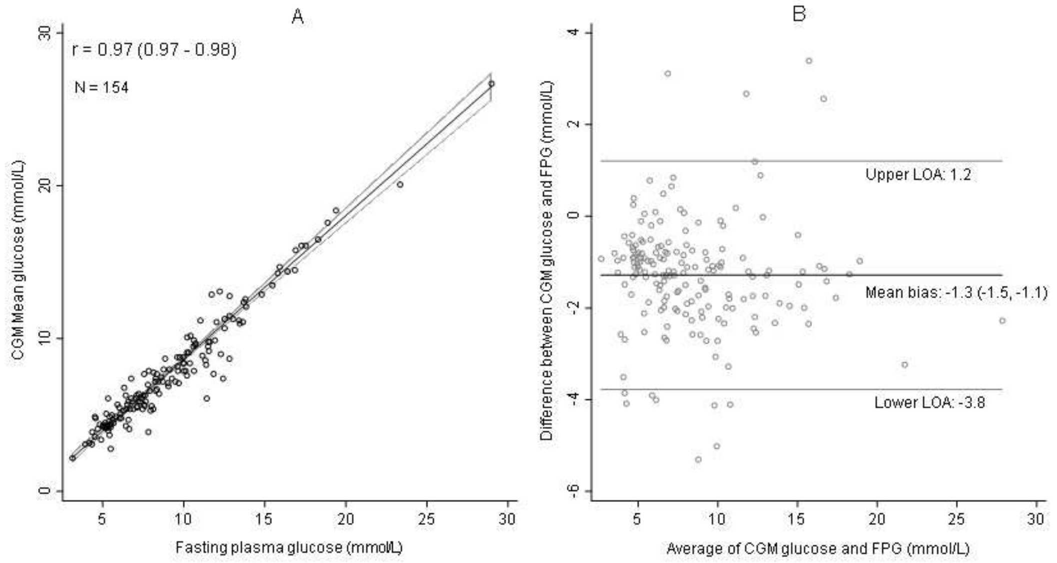

Figure S3: Comparison of CGM glucose and Fasting plasma glucose (A). A Bland Altman Plot of Fasting Plasma Glucose test and CGM sensor glucose. The black solid line denotes the mean bias between the fasting plasma glucose tests and average cgm glucose and the grey solid lines denote upper and lower limits of agreement (LOA). Overall CGM underestimated plasma glucose by 1.3 mmol/L, with LOA from ranging between − 3.8 to 1.2 mmol/L

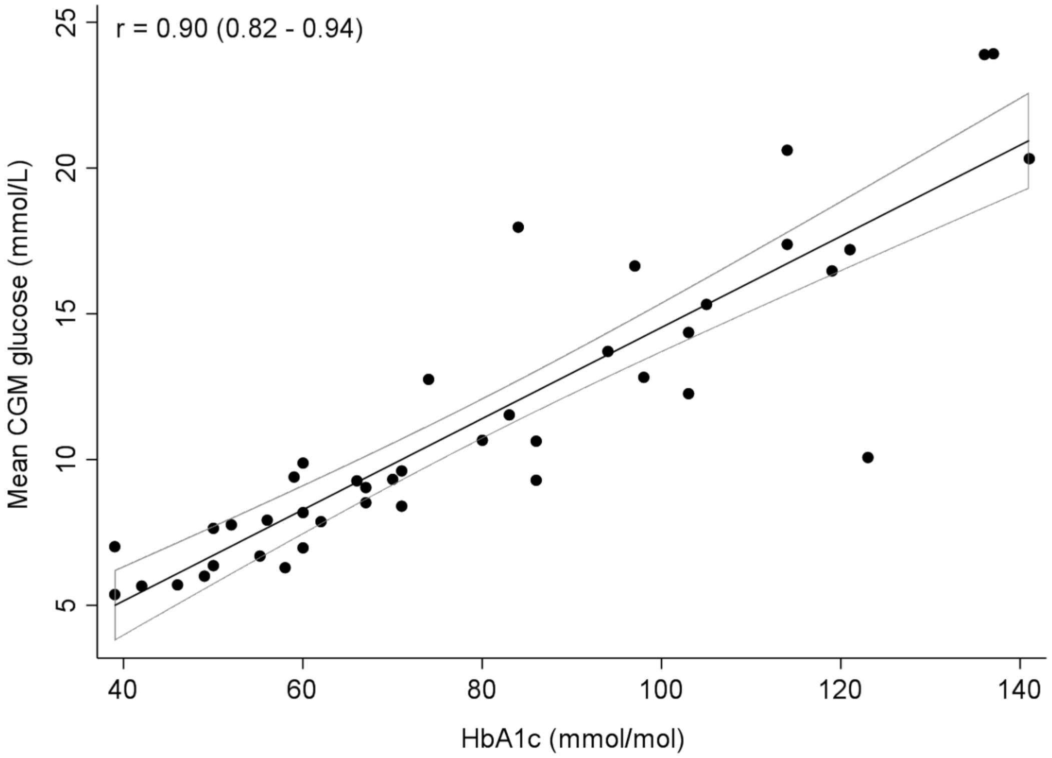

Figure S4: Comparison of HbA1c to mean CGM glucose among those with haemoglobinopathies. Dark thick straight line denotes the line of best fit and the thin lines represent the 95% confidence interval. The Pearson's correlation coefficient (r) and 95% confidence intervals are shown on the left upper corner of the graph.
