# Supplementary material for: HbA1c performs well in monitoring glucose control even in populations with high prevalence of medical conditions that may alter its reliability: the OPTIMAL observational multicenter study
Source: BMJ Open Diabetes Res Care. 2021 Sep 17;9(1):e002350. doi: 10.1136/bmjdrc-2021-002350 (PMC8451306; doi:10.1136/bmjdrc-2021-002350)
Supplement: Supplementary data [file bmjdrc-2021-002350supp002.pdf]

Supplementary Table 1: Participant characteristics of those included in the final analysis (n = 192) versus those excluded (n = 21)

	Median (IQR) for continuous variables, n (%) for proportions	
	Included	Not included
**Number, n (%)**	192 (90.1)	21 (9.9)
**Clinical**		
Female, n (%)	112 (58.3)	13 (61.9)
Age, years	56 (50, 63)	52 (48, 60)
Duration of diabetes, years	6 (3, 10)	7 (1, 11)
BMI, kg/m^2	26.8 (24.0, 30.5)	28.5 (27.6, 33.8)
**Current management n (%)**		
Metformin only	30 (15.6)	2 (9.5)
SU (+/- metformin)[a]	110 (57.3)	13 (61.9)
Insulin (+/- other diabetes drug)[b]	50 (26.0)	6 (28.6)
Diet [c]	2 (1.0)	0 (0.0)
**Glycaemia**		
HbA1c, %	8.3 (6.9, 10.0)	7.7 (6.0, 9.1)
HbA1c, mmol/mol	67 (52.0, 90.0)	61.0 (42.5, 76.0)
Fasting plasma glucose, mmol/L	8.2 (6.1, 11.4)	7.0 (5.8, 12.3)
Random plasma glucose, mmol/L	13.5 (8.8, 17.2)	10.8 (7.6, 17.2)
**Other laboratory**		
Hb (g/L)	14.2 (13.2, 15.0)	14.5 (14.1, 15.5)
eGFR	111.5 (92.3, 121.0)	117.8 (96.7, 124.7)

Supplementary Table 2: Participant characteristics presence (Group 2) or absence (Group 1) of HbA1c comorbidities

	Median (IQR) for continuous variables, % (n) for proportions	
**Clinical**	**Group 1**	**Group 2**
**N (%)**	**67.2 (129/192)**	**32.8 (63/192)**
Female, n (%)	60.5 (78/192)	54.0 (34/192)
Age, years	55 (50, 61)	58 (50, 64)
Duration of diabetes, years	6 (3, 10)	9 (4, 12)
BMI, kg/m^2	27. 1 (24.3, 30.3)	25.8 (23.1, 30.6)
**Current management n (%)**		
Metformin only	18.6 (24/129)	9.5 (6/63)
SU (+/- metformin)[a]	57.4 (74/129)	57.1 (36/63)
Insulin (+/- other diabetes drug)[b]	22.5 (29/129)	33.3 (21/63)
Diet [c]	2 (1.5)	0
**Glycaemia**		
CGM glucose, mmol/L	8.4 (6.8, 12.3)	9.3 (7.0, 12.3)
HbA1c, %	8.2 (6.7, 9.8)	8.7 (7.1, 10.7)
HbA1c, mmol/mol	66.0 (50.0, 85.0)	70.5 (54.0, 97.0)
Fasting plasma glucose, mmol/L	8.3 (6.1, 11.3)	7.8 (5.9, 11.5)
Random plasma glucose, mmol/L	13.0 (8.8, 16.8)	14.1 (8.7, 18.4)

Group 1 includes all those without HbA1c comorbidities (n = 129) and Group 2 includes all those with HbA1c comorbidities (n = 63). HbA1c comorbidities are the non-glycaemic biological conditions thought to alter HbA1c reliability e.g., haemo-globinopathies including sickle cell, anaemia, and renal impairment.

Supplementary Table 3: Glycaemic measures correlated with mean day-to-day glucose measured by CGM stratified by presence or absence of comorbidities thought to alter HbA1c reliability

	Overall	Group 1	Group 2
Fasting			
N	192	129	63
r (95% CI)	0.82 (0.76 – 0.86)	0.84 (0.78 – 0.89)	0.78 (0.67 – 0.86)
LR equation	Mean CGM = 0.91 (fasting) + 1.77	Mean CGM = 1.02 (fasting) + 0.71	Mean CGM = 0.77(fasting) + 3.13
Random			
N	192	129	63
R (95% CI)	0.76 (0.69 – 0.81)	0.74 (0.65 – 0.81)	0.80 (0.69 – 0.87)
LR equation	Mean CGM = 0.53(random) + 2.66	Mean CGM = 0.53(random) + 2.80	Mean CGM = 0.55(random) + 2.37
HbA1c			
N	192	129	63
R (95% CI)	0.88 (0.84 – 0.91)	0.89 (0.85 – 0.92)	0.85 (0.76 – 0.91)
LR equation	Mean CGM = 0.15(HbA1c) - 0.61	Mean CGM = 0.16(HbA1c) – 1.07	Mean CGM = 0.14(HbA1c) – 0.02

Group 1 includes all those without HbA1c comorbidities (n = 129) and Group 2 includes all those with HbA1c comorbidities (n = 63). Comorbidities are the non-glycaemic biological conditions thought to alter HbA1c reliability e.g., haemo-globinopathies including sickle cell, anaemia, and renal impairment.

Supplementary Table 4: Short-term glycaemic measures correlated with HbA1c stratified by presence or absence of comorbidities thought to alter HbA1c reliability

	Overall	Group 1	Group 2
Fasting			
N	208	142	66
r (95% CI)	0.70 (0.62 – 0.76)	0.78 (0.71 – 0.84)	0.57 (0.38 – 0.71)
LR equation	HbA1c = 4.62(fasting) + 29.55	HbA1c = 5.40(fasting) + 21.13	HbA1c = 3.57(fasting) + 41.92
Random			
N	211	145	66
r	0.74 (0.68 – 0.80)	0.74 (0.66 – 0.81)	0.74 (0.61 – 0.83)
LR equation	HbA1c = 3.09(random) + 29.01	HbA1c = 3.07 (random) + 28.58	HbA1c = 3.12(random) + 30.39

Group 1 includes all those without HbA1c comorbidities (n = 129) and Group 2 includes all those with HbA1c comorbidities (n = 63). Comorbidities are the non-glycaemic biological conditions thought to alter HbA1c reliability e.g., haemo-globinopathies including sickle cell, anaemia, and renal impairment.
